# Referral and access to care of HIV prevalent cases; experience from the early capture HIV cohort study in Kampala

**DOI:** 10.1186/1742-4690-9-S2-O28

**Published:** 2012-09-13

**Authors:** LN Mutengu, H Kibuuka, M Millard, A Sekiziyivu, S Wakabi, J Nanyondo, G Kawooya, M Robb, N Michael

**Affiliations:** 1 Makerere University Walter Reed Project, Kampala, Uganda; 2 US Military HIV Research Program , Rockridge, MD, USA

## Background

Trial sponsors and implementers are ethically obligated to refer HIV infected Individuals identified in a research study at screening for HIV care and treatment. Makerere University Walter Reed Project is conducting HIV surveillance among high risk uninfected female sex workers. We describe patterns in participants’ receipt of HIV results and response to referral for HIV care and treatment.

## Methods

Subsequent to informed consent, risk eligibility is determined using Audio Computer Assisted Self Interview (ACASI). Medical history, physical exam and blood draw are done to determine HIV sero-status and further eligibility. Participants determined HIV positive by ELISA/Western Blot require confirmatory testing before being screened out and referred for care.

## Results

HIV prevalence was 35% (221/631) at screening. Out of the 221 prevalent cases, only 96 participants (43%) received HIV confirmatory results and were referred for care, while 9(4%) declined referral. The majority did not return for either their initial or confirmatory HIV result; while a few declined a blood re-draw. Of the 96 participants referred, 58% are currently in care, 14% did not report for care predominately citing indecisiveness while 28% could not be tracked. Majority of acutely infected participants (6/8) are in care.

## Conclusion

Although trial implementers may fulfil their obligation in referring study participants for HIV care, participants have a key role to play in facilitating this process. The large number of HIV prevalent female sex workers who did not return for their HIV results and may not be aware of their status could be a potential driver of the epidemic in Uganda.

